# When Diagnosis Meets Reality: Operational Performance of Routine Malaria Diagnostics Against Molecular Evidence in Southwestern Saudi Arabia

**DOI:** 10.1155/jotm/8453252

**Published:** 2026-10-21

**Authors:** Musa A. Ayashi, Ahmed A. Muyidi, Majed H. Wakid, Fadi M. Baakdah, Maimonah S. Alghanmi, Hattan S. Gattan, Amro A. Zalah, Emad E. A. Wasili, Abdulaziz Y. Bajawi, Thamer M. Brek

**Affiliations:** ^1^ Main Laboratory and Blood Bank Medical and Molecular Microbiology Laboratory Jazan Health Cluster Jazan Saudi Arabia; ^2^ Main Laboratory and Blood Bank Medical and Molecular Microbiology Laboratory King Faisal Medical City for Southern Region Abha Saudi Arabia; ^3^ Department of Medical Laboratory Sciences Faculty of Applied Medical Sciences King Abdulaziz University Jeddah 21589 Saudi Arabia kau.edu.sa; ^4^ Special Infectious Agents Unit King Fahd Medical Research Center King Abdulaziz University Jeddah 21589 Saudi Arabia kau.edu.sa; ^5^ Vaccines and Immunotherapy Unit King Fahd Medical Research Center King Abdulaziz Aziz University Jeddah Saudi Arabia kau.edu.sa; ^6^ Molecular Biology Department Jazan Health Cluster Jazan Saudi Arabia; ^7^ Biotechnology Department Jazan Health Cluster Jazan Saudi Arabia; ^8^ Main Laboratory and Blood Bank Medical Microbiology Laboratory Jazan 86735 Saudi Arabia; ^9^ Main Laboratory and Blood Bank Medical Microbiology Laboratory Jazan Health Cluster Jazan Saudi Arabia

**Keywords:** diagnostic accuracy, light microscopy, malaria diagnosis, malaria elimination, nested PCR, operational performance, rapid diagnostic tests, Saudi Arabia

## Abstract

**Background:**

Saudi Arabia has reported no indigenous malaria since 2021 and is in the prevention‐of‐reintroduction phase, with residual and imported infections concentrated in Jazan and Aseer. At this stage, each reported case triggers programmatic action, yet the operational accuracy of routine facility diagnostics has not been measured in this setting.

**Objective:**

To evaluate the operational accuracy of facility‐performed routine light microscopy (RLM) and two Pf/PAN rapid diagnostic tests (RDTs) against species‐specific nested PCR.

**Methods:**

Retrospective diagnostic accuracy study on consecutively archived specimens. Of 118 archived EDTA samples from febrile patients tested for suspected malaria at hospitals in nine governorates of southwestern Saudi Arabia during January–December 2023, 106 were eligible (12 were excluded). Index tests were performed by facility staff during routine care; nested PCR was later performed blinded. Accuracy was assessed against nested PCR and, in a sensitivity analysis, a composite reference (RLM or PCR positive).

**Results:**

Nested PCR detected *Plasmodium* in 81/106 samples (76.4%; 95% CI 67.2–84.0), all single‐species (*P. falciparum* 56/81; *P. vivax* 25/81). RLM was positive in 98/106 (92.5%), CareStart in 106/106 (100%), QuickProfile in 100/106 (94.3%). RLM called 29/98 positives mixed (29.6%) versus 0/81 by PCR (Fisher’s exact, *p* < 0.001). Against PCR, RLM sensitivity was 97.5% (91.3–99.6) and specificity 24.0% (10.2–45.5); CareStart specificity 0% (0–13.9), QuickProfile 20.0% (8.1–39.8). PCR confirmation increased with reported parasitemia, from 12/23 (52.2%) at 10–90 parasites/μL to 22/24 (91.7%) at 1000–10,000 parasites/μL (Cochran–Armitage, *p* < 0.001).

**Conclusion:**

In the selected clinically suspected cases, routine diagnostics returned more positives, and far more mixed infections, than nested PCR; both routine false positives and reduced PCR sensitivity in archived material plausibly contribute. Findings should not be extrapolated beyond this population. Strengthened microscopy training, RDT quality assurance, and evaluation of molecular confirmation may support case classification during the prevention‐of‐reintroduction phase.

## 1. Background

Malaria transmission in southwestern Saudi Arabia has declined substantially over the past 2 decades, driven by national elimination efforts, yet focal transmission persists with coendemic *Plasmodium falciparum* and *P. vivax*, necessitating accurate diagnosis for effective case management and surveillance [1, 2].

The scale of that decline is documented in national surveillance: autochthonous cases fell from 511 in 2000 to 30 in 2014, and Saudi Arabia has subsequently reported no indigenous cases from 2021 onward, sustaining zero indigenous transmission for three consecutive years as of the 2024 World malaria report and moving from the elimination phase into prevention of reintroduction [3, 4]. The residual burden is now dominated by imported and occasional introduced infections, concentrated in Jazan and Aseer along the Yemeni border and sustained by population movement from malaria‐endemic countries in the Horn of Africa and South Asia [5, 6]. In this epidemiological context, the operational consequences of a diagnostic error change: Because indigenous transmission is at or near zero, a false‐positive result may be investigated as a potential focus of re‐establishment, while a false‐negative result in an imported case represents an onward transmission risk in a receptive area. Accurate case ascertainment is therefore no longer a purely clinical matter but the basis on which the elimination status of the country is judged.

Prospective studies in the region using controlled conditions reported microscopy‐based malaria prevalence of 0.5%–8% among febrile patients, with *P. falciparum* comprising 60%–85% of cases, *P. vivax* 15%–35%, and mixed infections 5%–30% [7, 8]. Evaluations of rapid diagnostic tests (RDTs) showed sensitivity of 85%–98% and specificity of 90%–99% against microscopy, while polymerase chain reaction (PCR) detected 1.5–3 times more infections, including mixed cases in 10%–40% of positives [9, 10]. However, these assessments involved research team oversight, not reflecting routine facility performance.

Nationally, Saudi Arabia’s malaria control program mandates microscopy and RDTs for diagnosis, with low overall prevalence (0.6%–0.7% in febrile cases), predominantly imported [3, 5]. Studies highlight RDT expansion but note limitations in low‐density detection, without retrospective evaluations of operational accuracy [11].

Internationally, operational studies of routine facility diagnostics versus molecular methods reveal discrepancies: in Tanzania, routine RDTs showed 6.5% positivity versus 2.9% microscopy and 4.2% qPCR, with high false positives [12]; in Ghana, routine RDT sensitivity was 55.7% and routine light microscopy (RLM) 39.3% against qPCR, missing submicroscopic infections [13]; and in India, facility microscopy and RDT detected 64% and 63% positives versus 76.5% by PCR [14]. Factors such as parasite density, genetic diversity (e.g., pfhrp2/3 deletions), storage conditions, operator errors, environmental factors (heat/humidity), host immunity, age, pregnancy, and prozone effects reduce the operational performance of RDTs and microscopy, leading to false positives/negatives [15–18]. Inaccurate diagnosis results in overtreatment (reported as high as 85% in some settings, although estimates vary widely with transmission intensity, case definition, and the reference standard used), drug resistance, delayed nonmalaria care, wasted resources, and side effects from unnecessary antimalarials like primaquine‐induced hemolysis, while inflating surveillance data and hindering control. Such operational assessments underscore gaps in field specificity and speciation, informing quality improvements.

Parasite genetic diversity has direct consequences for antigen‐detection diagnostics. HRP2‐based RDTs detect histidine‐rich protein 2 and, through shared epitopes, its paralog HRP3; *P. falciparum* lineages carrying deletions of *pfhrp2* and/or *pfhrp3* therefore escape detection and generate false‐negative results [19–21]. A global systematic review has documented these deletions across four continents with marked geographical heterogeneity [22], and WHO now reports them from more than 40 countries, although prevalence has reached the level requiring a change of RDT only in a smaller subset in South America and the Horn of Africa [23]. WHO recommends that national programs switch away from HRP2‐only RDTs once deletions causing false‐negative results exceed 5% of symptomatic *P. falciparum* infections [23]. Prevalence above that threshold have been reported in the Horn of Africa, including Eritrea, Djibouti, and Ethiopia, where dual *pfhrp2/3* deletion was found in 13.3% of genotyped isolates and in 70% of false‐negative RDT results in a recent multifacility survey [24]. This is directly relevant to southwestern Saudi Arabia, where a substantial share of the circulating parasite population is imported from these same source countries, even though deletions have been reported as rare among locally examined isolates [8]. Two implications bear on the interpretation of the present study. First, *pfhrp2/3* deletions cause false‐negative rather than false‐positive RDT results and therefore cannot account for excess RDT positivity. Second, genetic diversity is a two‐sided problem: sequence variation at primer‐binding sites, together with low‐density and minority‐species parasitemia, can reduce the sensitivity of nested PCR itself, so molecular methods must not be treated as infallible when they are used as a reference standard [25, 26].

Despite these insights, no studies in southwestern Saudi Arabia have retrospectively compared original routine facility‐reported microscopy and RDT results against reference nested PCR on archived samples, addressing operational performance in an elimination setting. This gap limits understanding of diagnostic accuracy under daily operational conditions, potentially inflating case estimates and guiding inappropriate treatment.

The present study aims to evaluate the operational concordance of routine malaria diagnostics (RLM and two Pf/PAN RDTs) performed by public health facilities in southwestern Saudi Arabia by comparing original facility results with species‐specific nested PCR on archived blood samples from the same febrile patients, providing novel evidence for surveillance and procurement decisions.

## 2. Materials and Methods

### 2.1. Study Design

This was a retrospective diagnostic accuracy study conducted on a consecutive cross‐section of archived clinical specimens. The term “retrospective cross‐sectional” is used here in a specific sense: each patient contributed a single set of concurrent test results obtained at one point in time (cross‐sectional), and all index testing had already been completed during routine clinical care before the study was conceived (retrospective). The index tests were RLM and two Pf/PAN RDTs performed and interpreted by facility staff during standard clinical care at public health facilities in southwestern Saudi Arabia. The reference standard was species‐specific nested PCR, performed subsequently and blinded on archived EDTA blood from the same patients. A single‐gate design was used: All participants were drawn from one clinical population, febrile patients in whom a clinician suspected malaria, and all received both the index tests and the reference standard, with no verification bias arising from selective referral to the reference test. No participant was contacted and no additional specimen was collected. Reporting follows the STARD 2015 statement for diagnostic accuracy studies [27].

### 2.2. Origin of the Specimens

The study area in southwestern Saudi Arabia has a population of approximately 1.57 million across 13,457 km^2^ and 16 governorates [28].

Sampling was by total enumeration of the archive rather than by selection. All 118 EDTA specimens held in the institutional archive for febrile patients evaluated for suspected malaria between January 2023 and December 2023 at hospitals across nine governorates of southwestern Saudi Arabia were screened for eligibility, and every specimen meeting the criteria was included, yielding 106 samples. Inclusion criteria were: (i) documented fever at presentation; (ii) clinician‐requested routine malaria testing using RLM and both RDT brands (CareStart HRP2 and QuickProfile Pf/Pan) as part of standard practice; and (iii) availability of ≥ 200 μL archived EDTA blood sufficient for nested PCR. Exclusion criteria were incomplete demographic or laboratory records (*n* = 6) and insufficient residual sample volume for PCR (*n* = 6). No specimen was excluded based on its routine test result, and no random or purposive subsampling was applied. Because the study analyzed the complete eligible archive for the period, no priori sample size calculation based on expected sensitivity and specificity was performed; the resulting precision, and in particular its consequences for the specificity estimates, is addressed in the Limitations (Figure 1).

**FIGURE 1:**
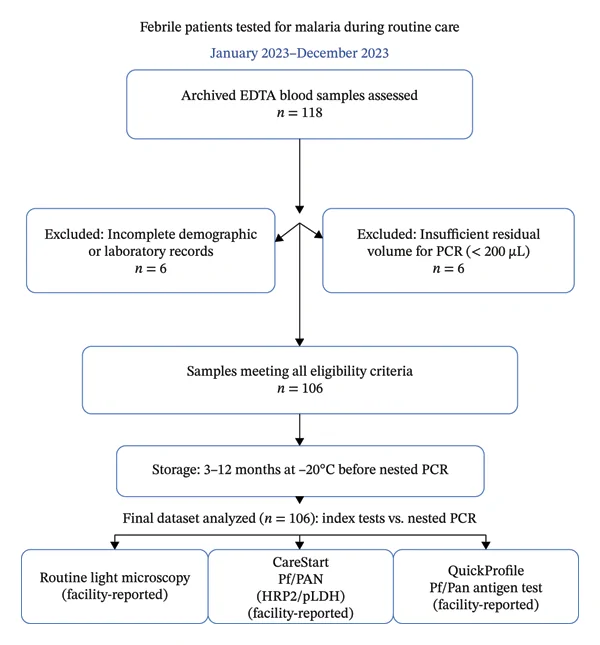
STARD flow diagram of specimen selection. Of 118 archived EDTA specimens from febrile patients tested for malaria during routine care between January and December 2023, 6 were excluded because of incomplete demographic or laboratory records and 6 because the residual volume was insufficient for PCR (< 200 μL), leaving 106 specimens meeting all eligibility criteria. For all included specimen, routine light microscopy, CareStart Pf/PAN and QuickProfile Pf/PAN results were retrievable from facility records and ≥ 200 μL of archived blood remained for nested PCR. Storage duration between routine testing and PCR ranged from 3 to 12 months.

The facility records available to the study team captured age, sex, nationality (Saudi/non‐Saudi), and rural or urban residence, but did not record the country of origin of non‐Saudi patients, travel history, date of arrival in Saudi Arabia, or duration of residence. Cases could therefore not be classified as imported, introduced, or indigenous from the study data, and no such classification was inferred. Likewise, RDT lot numbers, expiry dates, and storage temperature logs, and any prior antimalarial treatment, were not documented in the routine records. These gaps are intrinsic to the retrospective use of routine clinical documentation and are considered explicitly in the Limitations.

Because PCR was performed retrospectively, the time interval between routine diagnostic testing and PCR corresponded to the archive storage period, ranging from 3 to 12 months depending on the date of initial testing. All RLM and RDT procedures were performed by facility staff as part of standard care, with no involvement from the research team. Information on prior antimalarial treatment was not captured in the available facility records (Figure 1).

### 2.3. RLM Performed by Health Facilities

Thick and thin blood films were prepared, stained with 10% Giemsa (pH 7.2) for 10 min, and examined at 1000× magnification by facility laboratory staff according to standard national procedures. RLM parasitemia categories were defined in parasites/μL (e.g., 10–90 parasites/μL), based on facility‐reported estimates using standard Giemsa‐stained thick/thin films per WHO guidelines [29, 30]. At least 300 fields were examined before declaring a slide negative. Original results (positive/negative, species, mixed infections) were retrieved unchanged from facility records.

### 2.4. Routine RDTs Performed by Health Facilities

CareStart Malaria Pf/PAN (HRP2/pLDH) Ag Combo RDT and QuickProfile Malaria Pf/PAN Antigen Test were performed and interpreted by facility staff per manufacturer instructions and local protocols [31, 32]. Results were extracted from facility logbooks without research team intervention.

### 2.5. Deoxyribonucleic Acid Extraction and Nested PCR

Archived EDTA samples, stored at −20°C postroutine testing, were processed at the National Center for Vector‐Borne Diseases Laboratory. Genomic DNA was extracted using QIAamp DNA Blood Mini Kit (Qiagen) according to the manufacturer’s protocol [33]. Species‐specific nested PCR targeting *Plasmodium* 18S rRNA was performed for *P. falciparum*, *P. vivax*, *P. malariae*, and *P. ovale* (including *curtisi/wallikeri* differentiation) [10, 34]. *P. knowlesi* was not tested due to lack of primers. PCR was conducted blinded by an independent technician unaware of facility results (primer sequences and conditions in Table 1). Nested PCR served as the primary reference standard due to its superior sensitivity and speciation [25, 26]. A sensitivity analysis used a composite reference (positive if either microscopy or PCR positive).

**TABLE 1:** PCR primers specific for 18S RNA gene of *Plasmodium* spp.

Genus/species	Primer	Sequence 5′ ⟶ 3′	PCR bp	Denaturation (°C, min)	Annealing (°C, min)	Elongation (°C, min)	No. of cycles
*Plasmodium*	F	TTAAAATTGTTGCAGTTAAAACG	1200	(94, 1)	(58, 2)	(72, 2)	25
	R	CCTGTTGTTGCCTTAAACTTC					
*P. falciparum*	F	TTAAACTGGTTTGGGAAAACCAAATATATT	205				30
	R	ACACAATGAACTCAATCATGACTACCCGTC					
*P. vivax*	F	CGCTTCTAGCTTAATCCACATAACTGATAC	120				
	R	ACTTCCAAGCCGAAGCAAAGAAAGTCCTTA					
*P. malariae*	F	ATAACATAGTTGTACGTTAAGAATAACCGC	144				
	R	AAAATTCCCATGCATAAAAAATTATACAAA					
*P. ovale*	F	ATCTCTTTTGCTATTTTTTAGTATTGGAGA	800				
	R	GGAAAAGGACACATTAATTGTATCCTAGTG					

*P. ovale* (*curtisi* and *wallikeri*)	F	GCTGTAGCTAATACTTGCTTTA	877	(95, 1)	(55, 1)	(72, 1)	25
	R	TTCACCTCTGACATCTGAATC					

*Note:* F = forward; R = reverse; min = minute; bp = base pair.

### 2.6. Ethical Approval

This retrospective study analyzed archived samples without direct patient involvement. Approval was obtained from the Ethics Committee, Faculty of Applied Medical Sciences, King Abdulaziz University, Jeddah, Saudi Arabia (FAMS‐EC2021‐09).

### 2.7. Statistical Analysis

Data were entered in Microsoft Excel 2016 (Microsoft Corporation, Redmond, WA, USA) and analyzed in IBM SPSS Statistics for Windows, version 25.0 (IBM Corp., Armonk, NY, USA). Categorical variables are summarized as counts with percentages, and every percentage is reported with the numerator and denominator from which it was derived. The two continuous variables in the dataset, patient age and RLM‐reported parasite density, were available to the study team only as the predefined categories recorded in the routine registers rather than as exact values; means with standard deviations and medians with interquartile ranges could therefore not be computed for them, and they are summarized as counts and percentages within those categories. No other continuous variable was analyzed. Sensitivity, specificity, positive predictive value (PPV), negative predictive value (NPV), positive and negative likelihood ratios, and overall accuracy were calculated from 2 × 2 tables using nested PCR as the primary reference standard, each with exact (Clopper–Pearson) 95% confidence intervals; likelihood ratios and NPV are not estimable where a cell count is zero and are reported as such. Agreement between paired methods was quantified using Cohen’s κ with 95% CIs and interpreted using the Landis and Koch benchmarks (≤ 0.20 slight; 0.21–0.40 fair; 0.41–0.60 moderate; 0.61–0.80 substantial; > 0.80 almost perfect). The association between RLM‐reported parasitemia category and nested PCR confirmation was tested with the Cochran–Armitage test for trend across ordered density categories. Comparisons of proportions between independent groups, including the sex‐stratified comparisons and the comparison of mixed infection frequency between RLM and nested PCR, used Fisher’s exact test. All tests were two sided and an a priori significance level of α = 0.05 was set. No adjustment was made for multiple comparisons, and the sex‐stratified analyses are accordingly treated as exploratory. The *p* values are reported as below 0.001, to three decimal places between 0.001 and 0.01, and to two decimal places at or above 0.01. Missing data were not imputed, and all analyses were complete case.

## 3. Results

### 3.1. Study Population

A total of 106 febrile patients attending public health facilities in southwestern Saudi Arabia were included in the study. Males predominated (88/106, 83.0%), with 82.1% (87/106) being Saudi nationals, and residence evenly distributed between rural and urban settings (53/106 each). The largest age group was 18 to less than 30 years (50/106, 47.2%), followed by 30 to less than 40 years (26/106, 24.5%), less than 18 years (18/106, 17.0%), and 40 years or older (12/106, 11.3%) (Figure 2).

**FIGURE 2:**
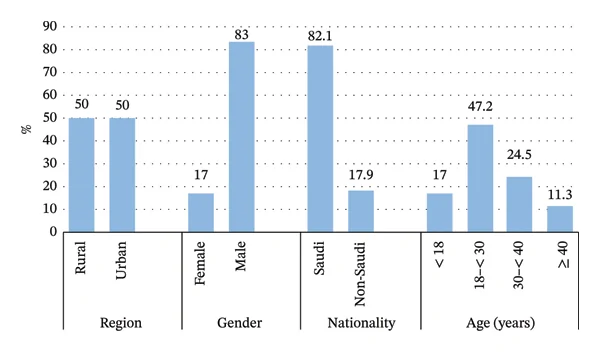
Demographic characteristics of the study population (*N* = 106). Distribution of the 106 febrile patients whose archived specimens were analyzed, by sex (male 88/106, 83.0%; female 18/106, 17.0%), nationality (Saudi 87/106, 82.1%; non‐Saudi 19/106, 17.9%), residence (rural 53/106, 50.0%; urban 53/106, 50.0%), and age group (< 18 years 18/106, 17.0%; 18 to < 30 years 50/106, 47.2%; 30 to < 40 years 26/106, 24.5%; ≥ 40 years 12/106, 11.3%). All percentages are of the total study population.

### 3.2. Malaria Detection and Species Identification

Reference nested PCR, performed on archived samples, detected *Plasmodium* DNA in 81 of 106 specimens (76.4%; 95% CI 67.2–84.0). All PCR‐detected infections were single‐species: *P. falciparum* in 56 of the 81 PCR‐positive samples (69.1% of PCR positives; 56/106, 52.8% of all specimens) and *P. vivax* in 25 of 81 (30.9% of PCR positives; 25/106, 23.6% of all specimens). No mixed infections and no *P. malariae* or *P. ovale* were identified by nested PCR, and *P. knowlesi* was not tested. Throughout the Results, species proportions are expressed as a percentage of the test‐positive samples for the method concerned, not of the whole study population, unless stated otherwise.

In contrast, RLM reported 98 positive results (98/106, 92.5%; 95% CI 85.7–96.7), comprising 55 *P. falciparum* (55/98, 56.1% of RLM positives), 14 *P. vivax* (14/98, 14.3%), and 29 mixed *P. falciparum* + *P. vivax* infections (29/98, 29.6%); the difference in mixed infection frequency between RLM (29/98) and nested PCR (0/81) was significant (Fisher’s exact test, *p* < 0.001). CareStart Malaria Pf/PAN RDT (RDT1) yielded positive results in every sample tested (106/106, 100%; 95% CI 96.6–100), comprising 5 Pf‐only (5/106, 4.7%), 11 Pan‐only (11/106, 10.4%), and 90 Pf + Pan (90/106, 84.9%). QuickProfile Malaria Pf/PAN RDT (RDT2) was positive in 100 of 106 samples (94.3%; 95% CI 88.0–97.8), comprising 12 Pf‐only (12/100, 12.0%), 17 Pan‐only (17/100, 17.0%), and 71 Pf + Pan (71/100, 71.0%) (Table 2).

**TABLE 2:** Detection and speciation by method.

Method	Positive *N* (%)	Pf *N* (%)	Pv/PAN *N* (%)	Mixed *N* (%)	Negative *N* (%)	Total *N* (%)
Nested PCR	81 (76.4)	56 (69%)	25 (30.9)	0 (0)	25 (23.6)	106
RLM	98 (92.5)	55 (56.1)	14 (14.3)	29 (29.6)	8 (7.5)	106
CareStart RDT1	106 (100)	5 (4.7)	11 (10.4)	90 (84.9)	0 (0)	106
QuickProfile RDT2	100 (94.3)	12 (12)	17 (17)	71 (71)	6 (5.7)	106

*Note:* For nested PCR and RLM, the “non‐*falciparum*” column corresponds to *P. vivax*; for RDTs, it corresponds to PAN‐line positivity (non‐*falciparum Plasmodium* spp.). Mixed infections for RDTs are defined as *Pf* + Pan. Pan = non‐*falciparum* and *Plasmodium* spp.

Abbreviations: LM = light microscopy, Pf = *P. falciparum*, Pv = *P. vivax*, RDT = rapid diagnostic test.

Cross‐tabulation of each routine test against nested PCR clarifies the direction of the discordance. Of the 98 RLM‐positive samples, 79 were confirmed by PCR and 19 were not; of the 8 RLM‐negative samples, 2 were PCR‐positive and 6 PCR‐negative. CareStart was positive in all 81 PCR‐positive and in all 25 PCR‐negative samples, producing 25 discordant positives and no negative results of any kind. QuickProfile was positive in 80 of 81 PCR‐positive and in 20 of 25 PCR‐negative samples, giving 20 discordant positives, 1 discordant negative, and 5 concordant negatives. The 19 RLM‐positive/PCR‐negative results were concentrated at low reported densities: 11 of 19 (57.9%) had been reported at 10–90 parasites/μL, 6 of 19 (31.6%) at 100–1000 parasites/μL, and 2 of 19 (10.5%) at 1000–10,000 parasites/μL.

### 3.3. Diagnostic Performance Using Nested PCR as the Reference Standard

When nested PCR served as the reference standard, RLM demonstrated high sensitivity (97.5%; 95% CI 91.3–99.6) but markedly reduced specificity (24.0%; 95% CI 10.2–45.5), resulting in 19 false‐positive diagnoses (PPV 80.6%; 95% CI 71.4–87.4; NPV 75.0%; 95% CI 40.9–92.9). CareStart RDT1 exhibited 100% sensitivity (95% CI 95.5–100) but 0% specificity (PPV 76.4%; 95% CI 67.2–84.0; NPV not calculable). QuickProfile RDT2 achieved sensitivity of 98.8% (95% CI 93.5–99.9) and specificity of 20.0% (95% CI 8.1–39.8; PPV 80.0%; 95% CI 70.9–87.0; NPV 83.3%; 95% CI 43.7–96.9) (Table 3).

**TABLE 3:** Performance of diagnostic methods vs. nested PCR.

Performance	RLM	CareStart RDT1	QuickProfile RDT2
Sensitivity % (95% CI)	97.5 (91.3–99.6)	100 (95.5–100)	98.8 (93.5–99.9)
Specificity % (95% CI)	24.0 (10.2–45.5)	0 (0–13.9)	20.0 (8.1–39.8)
PPV % (95% CI)	80.6 (71.4–87.4)	76.4 (67.2–84.0)	80.0 (70.9–87.0)
NPV % (95% CI)	75.0 (40.9–92.9)	—	83.3 (43.7–96.9)
+LR (95% CI)	1.28 (1.07–1.54)	1.00 (1.00–1.00)	1.24 (1.07–1.43)
–LR (95% CI)	0.10 (0.02–0.43)	—	0.06 (0.01–0.43)
*κ* (95% CI)	0.28 (0.11–0.46)	< 0.001	0.25 (0.05–0.46)

*Note:* For RDT1, NPV and likelihood ratios are not estimable because there were no test‐negative results and specificity = 0; these cells are reported as “–”. κ = Cohen’s kappa; +LR = positive likelihood ratio; –LR = negative likelihood ratio.

Abbreviations: CI = confidence interval, NPV = negative predictive value, PPV = positive predictive value.

These specificity estimates rest on a small number of reference‐negative samples and are correspondingly imprecise, as the width of their confidence intervals indicates: the CareStart point estimate of 0% has an upper 95% confidence limit of 13.9%, and the RLM estimate of 24.0% is compatible with true values between 10.2% and 45.5%. The 19 RLM and 25 CareStart discordant positives are described here relative to nested PCR; the extent to which they represent routine false positives rather than false‐negative PCR results in archived material is examined in the Discussion.

Pairwise agreement (Cohen’s *κ*) with nested PCR was fair for RLM (*κ* = 0.281; 95% CI 0.106–0.456) and QuickProfile RDT2 (*κ* = 0.254; 95% CI 0.045–0.463), and absent for CareStart RDT1 (*κ* < 0.001).

### 3.4. Sensitivity Analysis Using a Composite Reference Standard

Using a composite reference standard (defined as positive if either RLM or nested PCR was positive), 98 true malaria cases were identified (prevalence 92.5%; 95% CI 85.7–96.7). Against this composite standard, nested PCR specificity was 75.0% (95% CI 46.8–91.1; sensitivity 82.7%; 95% CI 73.9–89.1), CareStart RDT1 specificity was 0% (sensitivity 100%; 95% CI 96.3–100), and QuickProfile RDT2 achieved 100% sensitivity (95% CI 96.3–100) and 75.0% specificity (95% CI 46.8–91.1), with almost perfect agreement (*κ* = 0.847; 95% CI 0.669–1.000) (Table 4).

**TABLE 4:** Performance of diagnostic methods versus composite reference in the 106 tested samples.

Performance	Nested PCR	CareStart RDT1	QuickProfile RDT2
Sensitivity % (95% CI)	82.7 (73.9–89.1)	100 (96.3–100)	100 (96.3–100)
Specificity % (95% CI)	75 (46.8–91.1)	0 (0–41.0)	75 (46.8–91.1)
PPV % (95% CI)	98.8 (93.5–99.9)	92.5 (85.7–96.7)	98 (93.0–99.5)
NPV % (95% CI)	24.0 (10.2–45.5)	—	100 (54.1–100)
+LR (95% CI)	3.31 (1.50–7.31)	1 (1.00–1.)	4 (1.82–8.81)
–LR (95% CI)	0.23 (0.15–0.36)	—	0 (0–∞)
*κ* (95% CI)	0.28 (0.106–0.456)	< 0.001	0.85 (0.669–1)

*Note:* Composite reference = positive if RLM or nested PCR positive; nested PCR is included to estimate its false negatives against the composite. +LR = positive likelihood ratio; –LR = negative likelihood ratio; κ = Cohen’s kappa.

Abbreviations: CI = confidence interval, NPV = negative predictive value, PPV = positive predictive value.

The contrast between QuickProfile agreement against nested PCR (*κ* = 0.254) and against the composite reference (*κ* = 0.847) follows from the construction of the two standards rather than from any change in the behavior of the test. The composite standard classifies a sample as positive if either RLM or PCR is positive and therefore contains 98 positives and 8 negatives; because QuickProfile was positive in 100 samples, its results align closely with that marginal distribution and κ rises. Against nested PCR, with 81 positives and 25 negatives, the identical set of QuickProfile results yields substantial disagreement. Cohen’s κ is known to be sensitive to the prevalence defined by the reference standard, and the composite analysis is reported here only as a prespecified sensitivity analysis. Because the composite standard incorporates RLM, one of the index tests, it is subject to incorporation bias and inflates the apparent performance of RLM and of any test that agrees with it; its estimates are therefore not unbiased measures of accuracy and are discussed further in the Limitations.

### 3.5. Association Between RLM Parasitemia and Nested PCR Confirmation

Parasite density reported by RLM was strongly associated with nested PCR positivity (Cochran–Armitage trend test, *p* < 0.001). Nested PCR confirmed 22 of 24 samples (91.7%) reported at 1000–10,000 parasites/μL and 3 of 3 (100%) reported above 10,000 parasites/μL, compared with 12 of 23 (52.2%) reported at 10–90 parasites/μL, and 2 of 8 (25.0%) reported as negative by microscopy. This gradient is consistent with overcalling of low‐density films by microscopy, with loss of PCR sensitivity at low parasite densities, or with a combination of the two (Table 5).

**TABLE 5:** RLM parasitemia vs. PCR confirmation.

RLM (parasites/μL)	Positive nested PCR *N* (%)	Negative nested PCR *N* (%)	Total
0	2 (25.0)	6 (75.0)	8
1–10	1 (100)	0 (0)	1
10–90	12 (52.2)	11 (47.8)	23
100–1000	41 (87.2)	6 (12.8)	47
1000–10,000	22 (91.7)	2 (8.3)	24
> 10,000	3 (100)	0 (0)	3
Total	81 (76.4)	25 (23.6)	106

*Note:* PCR = nested PCR. Percentages are row percentages, i.e., the proportion of samples in each RLM parasitemia category that were positive or negative by nested PCR; the Total row gives column percentages of all 106 specimens. The eight samples in the 0 parasites/μL category were reported as negative by RLM.

Abbreviation: RLM = routine light microscopy.

Cochran–Armitage trend test showed a significant positive trend in PCR positivity across increasing RLM parasitemia categories (*p* < 0.001).

### 3.6. Gender‐Stratified Diagnostic Performance

With RLM as comparator, nested PCR was negative in 4 of the 4 RLM‐negative samples from female patients (100%) and in 2 of the 4 from male patients (50%), a difference that was not statistically significant (Fisher’s exact test, *p* = 0.43). Nested PCR sensitivity relative to RLM was 12/14 (85.7%) in females and 67/84 (79.8%) in males (*p* > 0.99), and QuickProfile specificity was 3/4 (75.0%) in both sexes (*p* > 0.99). CareStart was positive in every sample from both sexes. No sex‐stratified difference reached statistical significance. These strata are very small, 18 females and 88 males, and only 8 RLM‐negative samples in the whole series, divided 4 and 4 between the sexes, so each sex‐specific specificity estimate rests on four observations and carries a confidence interval spanning most of the possible range. The comparisons were not prespecified and were not adjusted for multiplicity; they are reported for completeness and are hypothesis‐generating at best (Table 6).

**TABLE 6:** Sex‐stratified diagnostic performance (RLM as comparator).

Method	Gender	Sensitivity % (*n*/*N*)	Specificity % (*n*/*N*)	PPV % (*n*/*N*)	NPV % (*n*/*N*)
Nested PCR	Female	85.7 (12/14)	100 (4/4)	100 (12/12)	66.7 (4/6)
	Male	79.8 (67/84)	50.0 (2/4)	97.1 (67/69)	10.5 (2/19)

CareStart RDT1	Female	100 (14/14)	0 (0/4)	77.8 (14/18)	NE
	Male	100 (84/84)	0 (0/4)	95.5 (84/88)	NE

QuickProfile RDT2	Female	100 (14/14)	75.0 (3/4)	93.3 (14/15)	100 (3/3)
	Male	100 (84/84)	75.0 (3/4)	98.8 (84/85)	100 (3/3)

Total samples	Female	18	—	—	—
	Male	88	—	—	—

Overall total		106	—	—	—

*Note:* RLM is the comparator throughout this table, so “sensitivity” denotes the proportion of RLM‐positive samples that the stated method also called positive, and “specificity” the proportion of RLM‐negative samples it called negative. Denominators are small in the RLM‐negative strata (4 females and 4 males), and NPV is not estimable (NE) for CareStart because that test returned no negative result. Female versus male comparisons by Fisher’s exact test: specificity of nested PCR, 4/4 versus 2/4, *p* = 0.43; sensitivity of nested PCR, 12/14 versus 67/84, *p* > 0.99; specificity of QuickProfile, 3/4 versus 3/4, *p* > 0.99. No sex‐stratified difference was statistically significant.

Abbreviations: NPV = negative predictive value, PPV = positive predictive value, RLM = routine light microscopy.

## 4. Discussion

The present study provides the first retrospective operational evaluation of malaria diagnostics used in public health facilities in southwestern Saudi Arabia. By comparing RLM and RDT results with reference‐nested PCR performed on archived specimens from the same febrile patients, we addressed an important evidence gap in elimination settings. Although controlled prospective studies dominate literature [3, 7, 8, 10, 30], their optimized conditions standardized training, close supervision, and regulated reagent handling, do not reflect routine operational performance. In contrast, operational settings involve variable staff capacity, heavy workloads, inconsistent storage conditions, and limited quality assurance. These realities were evident in our findings, which collectively reveal substantial overdiagnosis by RLM, poor concordance in mixed infection detection, heterogeneous RDT performance, parasitemia‐dependent confirmation rates, and gender‐related diagnostic variation. Together, these observations have direct implications for case management and surveillance reliability within Saudi Arabia’s malaria elimination program.

In our study, RLM reported a positive rate of 92.5% (98/106), exceeding nested PCR by 16.1% points, with 19 samples positive by RLM and negative by PCR. Such inflation stands in contrast to lower positivity rates documented in prospective regional studies (0.5%–8%) [7, 8], likely reflecting the improved accuracy achievable under supervised research conditions. National surveillance data similarly reported low malaria prevalence [5], supporting the inference that operational RLM may inflate case counts in practice. Similar tendencies toward overdiagnosis have been observed in other low‐transmission regions, such as Ghana and India [13, 14]. Misinterpretation of artifacts or low‐density infections likely contributes to this pattern [15, 35]. While microscopy sensitivity in our study (97.5%) aligns with previous controlled evaluations [8, 10], the markedly low specificity (24.0%) far below the 90%–99% range reported in research settings [3, 36], highlights the operational limitations exposed by our retrospective design.

An important caveat governs the interpretation of those 19 discordant results, and of the 25 discordant CareStart positives. The present design cannot separate true routine false positives from false‐negative nested PCR results with certainty, because no independent tie‐breaking assay was applied: archived films and RDT strips were not retained for blinded re‐reading, no quantitative or ultrasensitive PCR was performed, and no antigen quantification was undertaken. Three observations nonetheless bear on the balance of explanations. First, discordance was concentrated at the low end of reported parasitemia, with 11 of the 19 RLM‐positive/PCR‐negative samples reported at 10–90 parasites/μL, the density range in which both microscopy misreading and molecular false negatives are most likely. Second, samples had been stored at −20°C for 3–12 months before extraction, and DNA integrity in whole blood declines measurably over such intervals, so some loss of PCR sensitivity is to be expected [37, 38]. Third, HRP2 antigenemia persists for a median of approximately 15 days, and in a minority of patients for more than a month, after successful treatment [39], which can generate genuinely antigen‐positive but parasite‐negative samples; because prior antimalarial treatment was not recorded in the routine documentation, this mechanism could not be excluded. The most defensible reading of these data is therefore one of substantial discordance between routine and molecular diagnosis, in which routine overdiagnosis is the leading but not the sole explanation.

Nested PCR identified *P. falciparum* and *P. vivax* at proportions consistent with prior regional molecular studies, where *P. falciparum* typically predominates (60%–85%) and *P. vivax* accounts for 15%–35% [7, 8, 10, 34]. However, both RLM and RDTs diverged substantially from PCR in species allocation: microscopy underdetected *P. vivax* and substantially overcalled mixed infections, while RDTs reported disproportionately high Pf + Pan combinations. This mirrors earlier findings from the region indicating overrepresentation of non‐*falciparum* species by routine diagnostics [8]. Such distortions can affect treatment decisions, particularly given Saudi Arabia’s reliance on primaquine for *P. vivax* and mixed cases [5], a regimen that may cause hemolysis in G6PD‐deficient individuals, who represent approximately 8.4% of Saudi males in Jazan [40]. Globally, *P. vivax* predominance varies widely, particularly in Asia (50%–70%) [14, 41], underscoring the critical need for molecular confirmation to support accurate case classification and appropriate therapy in co‐endemic areas [42–44].

Speciation discrepancies were most striking in the detection of mixed infections. Although RLM identified 29.6% of cases as mixed *P. falciparum* + *P. vivax*, nested PCR detected none. Prospective regional PCR studies have documented mixed infections in 10%–40% of cases [3, 10, 34, 36, 44], indicating that neither microscopy nor PCR in this study fully captured expected patterns microscopy likely overcalling and PCR underdetecting. Long‐term storage of EDTA blood (up to 1 year at 4°C) presents a plausible explanation for reduced PCR sensitivity, particularly for low‐density minority species [37, 38]. Similar degradation effects have been reported where DNA integrity declines substantially in samples stored beyond 6 months [37]. qPCR, by contrast, offers substantially higher sensitivity and superior detection of mixed infections (up to 100% vs. 68% for nested PCR) [25, 26, 30, 45]. Despite its advantages, qPCR remains underutilized in the region [3, 8], yet represents a promising avenue for improving malaria surveillance accuracy, particularly in near‐elimination contexts [46–48].

Two mechanisms deserve particular emphasis in explaining how microscopy identified 29 mixed infections where PCR identified none. Microscopic differentiation of *P. vivax* from *P. falciparum* depends on morphological features that are degraded in thick films, and staining artefacts, superimposed platelets, and ring forms of a single species at differing maturity are all recognized sources of spurious mixed infection reports, particularly where slides are read under workload pressure without routine cross‐checking [15, 35]. Conversely, nested PCR detects a minority species poorly when it constitutes a small fraction of total parasite biomass, because the dominant species preferentially consumes primers and template during the genus‐level first round; sequential rather than simultaneous species amplification, combined with any degradation of template during storage, would be expected to remove precisely the low‐density second species that defines a mixed infection [25, 26, 45]. The true frequency of mixed infection in this population therefore probably lies between the two estimates rather than at either extremes, and resolving it will require quantitative species‐specific assays applied to freshly collected material.

RDTs performance differed considerably by brand. CareStart RDT1 returned a universal positivity rate (100%), resulting in a specificity of 0%. This implausible pattern suggests systematic false positives, potentially attributable to heat/humidity exposure, faint‐line misinterpretation, or HRP2 antigen persistence [15, 16]. In contrast, QuickProfile RDT2 performed better, achieving a specificity of 20% against PCR and 75% against RLM in the composite analysis, with high agreement with RLM (*κ* = 0.847). These findings not only align with controlled regional studies demonstrating RDT sensitivity of 85%–98% [3, 8], but also highlight how vulnerable RDTs are to operational conditions. Although pfhrp2/3 deletions can cause diagnostic failures [19–21], such deletions are rare in Saudi Arabia [8]. Externally, RDT performance varies broadly (specificity 20%–99%) depending on environmental conditions and parasite genetics [12, 13, 45, 49–51]. Given their central role in Saudi malaria diagnosis, routine monitoring of RDT storage and performance is essential, particularly in peripheral settings where conditions are difficult to control.

The universal positivity of CareStart warrants particular caution. All 106 strips were reported positive by facility staff, including in the 25 samples in which no *Plasmodium* DNA was detected, and the retrospective design precluded prospective investigation of this finding: used cassettes are not retained after reading, so independent re‐reading by trained investigators was not possible, and lot numbers, expiry dates, and storage temperature records were absent from the routine documentation. Several mechanisms are plausible and are not mutually exclusive. Persistent HRP2 antigenemia following recent treatment would produce genuine antigen positivity in the absence of viable parasites [39]. Exposure of cassettes to heat and humidity, difficult to control in peripheral facilities in this region, is a recognized cause of nonspecific line development, as is over‐reading of very faint lines when the operator expects a positive result in a patient referred with suspected malaria. Deletions of *pfhrp2/pfhrp3*, by contrast, cause false‐negative rather than false‐positive results [19–22, 24] and cannot explain this pattern. A specificity of 0% should accordingly be read as a signal that this brand, as used in these facilities under these conditions, provided no discriminatory information on this sample, rather than as a stable estimate of the intrinsic performance of the device. The finding requires prospective confirmation with retained strips, documented lot and storage data, and independent double reading before any procurement inference is drawn.

Comparisons with published operational evaluations should respect differences in transmission intensity. The comparisons drawn above with Ghana and Tanzania [12, 13] are informative about the general direction of routine‐versus‐molecular discordance, but those studies were conducted in moderate‐to‐high transmission settings in which a large reservoir of submicroscopic infection inflates PCR positivity relative to routine testing, the opposite of the pattern observed here. Settings closer to our own are more instructive. In Yemen, immediately across the border and the principal source of imported infection in Jazan, field evaluation of light microscopy, and a PfHRP‐2/pLDH RDT against nested PCR during peak seasonal transmission demonstrated the same problem of imperfect operational specificity under routine field conditions [52]. In southern Ethiopia, another important source of imported cases, facility‐based evaluation of an HRP2‐based RDT against PCR yielded a sensitivity of 76.5%, with discordance driven largely by *pfhrp2/3* deletion, a mechanism that reduces rather than increases RDT positivity, and which therefore cannot explain our findings, but which illustrates how strongly local parasite genetics shape routine performance [24]. Within the Arabian Peninsula itself, a study‐conditions comparison of RDT, microscopy, and nested PCR in 400 febrile patients in Jazan reported specificity above 99% for both microscopy and RDT against PCR [8]. The contrast between that figure and our operational estimates, obtained in the same region, reinforces the central argument of this analysis: the gap lies less in the tests themselves than in the conditions under which they are used. Differences between our results and those from high‐transmission settings should therefore be attributed to epidemiology and study conditions as much as to diagnostic quality.

The association between parasitemia and PCR confirmation further reinforces the limitations of RLM. Confirmation rates exceeded 91% for samples with ≥ 1000 parasites/μL, compared with only 52.2% among those with 10–90 parasites/μL, indicating a tendency for microscopy to overcall low‐density infections. Similar trends have been observed locally and internationally [8, 13, 46]. Such misclassification may inflate surveillance indicators, complicating elimination efforts. Cross‐border studies also show that submicroscopic infections missed by RLM and RDTs but detected by PCR are increasingly important in low‐transmission settings [13, 14, 53], highlighting the value of molecular confirmation [40].

Sex‐stratified analysis showed nested PCR to be negative in all 4 RLM‐negative samples from female patients but in only 2 of 4 from male patients. We initially read this as a sex difference in specificity, but on formal testing, the comparison is not significant (Fisher’s exact test, *p* = 0.43), and with four observations per stratum, it could not plausibly have been. We therefore make no claim of a sex difference in diagnostic performance in this dataset. Sex‐based differences in malaria parasite clearance and in immune response have been described elsewhere and remain biologically plausible [54–56], but the present data neither support nor exclude them, and invoking hormonal or immunological mechanisms to explain a nonsignificant difference between two four‐sample strata would be unwarranted. The wider point that survives is methodological rather than biological: subgroup analyses of diagnostic accuracy require stratum‐specific sample sizes that this study, designed around a fixed archive, could not provide, and adequately powered evaluation of demographic modifiers would need to be planned prospectively [57].

Collectively, these findings demonstrate that routine diagnostics face multiple challenges under operational conditions. Strengthening diagnostic performance will require targeted interventions such as selecting robust RDT brands (e.g., QuickProfile), enhancing RLM training particularly for low‐parasitemia and mixed species identification, and integrating molecular confirmation methods into routine surveillance. These priorities align with global calls for improved operational research to close the gap between controlled study performance and routine operational reliability [47, 48]. Emerging technologies such as AI‐assisted microscopy and digital RDT readers [58, 59] may further support accuracy by reducing observer variability.

## 5. Limitations

This study has several limitations, most of which follow from its retrospective design.

### 5.1. Spectrum and Selection Bias

Participants were febrile patients in whom a clinician had already suspected malaria and requested testing. This single‐gate design produced a PCR prevalence of 76.4% (81/106), far above the prevalence of malaria among unselected febrile patients in this region, and reflects the selection of highly suspected cases rather than community transmission intensity. Diagnostic accuracy estimates derived from such a sample are not transportable to unselected febrile patients: enrichment for true positives inflates sensitivity, and by leaving only 25 reference‐negative samples, it renders every specificity estimate imprecise. All conclusions are restricted to this clinically suspected population.

### 5.2. Sample Size and Precision

Because the study analyzed the complete eligible archive for the study period, no priori sample size calculation based on expected sensitivity and specificity was performed. With 25 reference‐negative samples, the 95% confidence intervals around specificity span tens of percentage points, and the CareStart point estimate of 0% is compatible with true values up to 13.9%.

### 5.3. Incorporation Bias in the Composite Reference Standard

Defining a case as positive if either RLM or nested PCR was positive incorporates one of the index tests into the reference standard. This is a recognized source of incorporation bias and necessarily inflates the apparent performance of RLM and of any test that agrees with it, which is why the composite analysis is reported only as a prespecified sensitivity analysis alongside the primary nested PCR standard rather than as an unbiased measure of accuracy.

### 5.4. Imperfection of the Reference Standard

Nested PCR is not a perfect reference. Archived EDTA blood stored at −20°C for 3–12 months may undergo DNA degradation, and nested PCR is relatively insensitive to a minority species within a mixed infection. Both mechanisms generate false‐negative PCR results and would inflate the apparent overdiagnosis attributed to routine methods. Neither quantitative PCR nor an independent tie‐breaking assay was available to adjudicate discordant results.

### 5.5. Unavailable Operational and Epidemiological Data

Routine records did not contain RDT lot numbers, expiry dates, or storage temperature logs, nor any record of prior antimalarial treatment, so kit‐level quality failures, environmental exposure, and persistent HRP2 antigenemia could not be evaluated as causes of the observed positivity. Records also lacked the country of origin, travel history, and duration of residence of non‐Saudi participants, so infections could not be classified as imported, introduced, or indigenous, and the relationship between the origin of infection and the observed species distribution could not be examined.

### 5.6. Absence of Independent Re‐Reading

Blood films and RDT cassettes were not retained after routine reporting, so no blinded re‐examination by reference microscopists or independent RDT readers was possible. Observer‐level sources of error, including faint‐line over‐reading and morphological misclassification of species, therefore cannot be quantified from these data.

### 5.7. Exploratory Subgroup Analysis

The sex‐stratified comparisons were not prespecified, were not adjusted for multiplicity, and rest on strata far too small to support inference: only 8 samples in the whole series were RLM‐negative, divided 4 and 4 between the sexes, so each sex‐specific specificity estimate is calculated on four observations. No sex‐stratified difference was statistically significant, and the analysis is reported for completeness rather than as evidence of a demographic effect.

These limitations do not negate the central observation of substantial discordance between routine and molecular diagnosis, but they define how far it can be interpreted. Apportioning that discordance definitively would require prospective evaluation using fresh specimens, quantitative PCR, retention of films and strips for blinded re‐reading, documented RDT storage and lot data, recorded treatment histories, and latent class analysis to accommodate an imperfect reference standard.

## 6. Conclusion

Among febrile patients with clinically suspected malaria whose specimens were archived in southwestern Saudi Arabia during 2023, RLM and two Pf/PAN RDTs identified more infections, and substantially more mixed infections, than species‐specific nested PCR performed on the same archived material. Within this selected population, the routine tests retained high sensitivity but showed low specificity against the molecular reference, and the discordance was concentrated at low reported parasite densities. These findings describe the performance of these particular tests, as used by these facilities, in this population. They are not an estimate of malaria prevalence in the region, and they should not be generalized to unselected febrile patients or to malaria diagnosis nationally. The study did not assess treatment decisions, patient outcomes, or programmatic case classification, and no inference about those endpoints is drawn from it.

What the findings do support is a case for strengthening quality assurance at the point where diagnosis is made: refresher training and routine cross‐checking for microscopists, with particular attention to low‐density films and to the criteria for reporting mixed infection; documented monitoring of RDT storage conditions, lot performance, and expiry dates in peripheral facilities; and retention of films and test cassettes to permit external re‐reading as part of a quality‐assurance cycle.

The role of molecular confirmation merits formal evaluation rather than immediate adoption. Nested PCR as applied here was itself constrained by the use of archived material and by limited sensitivity to minority species, and quantitative PCR was not part of this study’s methodology; the case for qPCR therefore rests on published comparisons reporting superior detection of low‐density and mixed infections [25, 26, 30, 45], not on the present data. A prospective study comparing routine diagnostics with qPCR on freshly collected specimens, with documented treatment histories and retained films and strips, would establish whether molecular confirmation adds sufficient value to justify its cost in a prevention‐of‐reintroduction setting such as southwestern Saudi Arabia.

## Author Contributions

M.H.W.: conceptualization, methodology, supervision, project administration, funding acquisition, resources, writing–original draft, writing–review and editing. M.A.A.: investigation, resources, data curation. A.A.M.: conceptualization, methodology, writing–original draft, writing–review and editing. F.M.B.: methodology, formal analysis. M.S.A.: methodology, validation. H.S.G.: formal analysis, validation. A.A.Z.: investigation, data curation. E.E.A.W.: investigation, data curation. A.Y.B.: investigation, data curation. T.M.B.: data curation. All authors contributed to the interpretation of the data and critically revised the manuscript for important intellectual content.

## Funding

This research received no specific grant from any funding agency in the public, commercial, or not‐for‐profit sectors.

## Disclosure

All authors read and approved the final version and agree to be accountable for all aspects of the work.

## Ethics Statement

This retrospective study analyzed archived clinical specimens without direct patient involvement, and no additional specimen was collected for research purposes. Ethical approval was granted by the Ethics Committee, Faculty of Applied Medical Sciences, King Abdulaziz University, Jeddah, Saudi Arabia (FAMS‐EC2021‐09). Because the study used previously collected, de‐identified archived material, the requirement for individual informed consent was waived by the Committee.

## Consent

The manuscript contains no data, images, or details relating to an identifiable individual.

## Conflicts of Interest

The authors declare no conflicts of interest.

## Data Availability

The datasets generated and analyzed during this study are not publicly available because they derive from routine clinical records held by the participating health facilities, but are available from the corresponding author on reasonable request and subject to the approval of the Jazan Health Directorate.
